# A generic theory of change-based framework with core indicators for monitoring the effectiveness of large-scale food fortification programs in low- and middle-income countries

**DOI:** 10.3389/fnut.2023.1163273

**Published:** 2023-06-22

**Authors:** Santiago Rodas-Moya, Francesca M. Giudici, Adedotun Owolabi, Folake Samuel, Stephen R. Kodish, Carl Lachat, Taymara C. Abreu, Karin H. van het Hof, Saskia J. M. Osendarp, Inge D. Brouwer, Edith J. M. Feskens, Alida Melse-Boonstra

**Affiliations:** ^1^Division of Human Nutrition and Health, Wageningen University & Research, Wageningen, Netherlands; ^2^Department of Human Nutrition and Dietetics, College of Medicine, Faculty of Public Health, University of Ibadan, Ibadan, Oyo State, Nigeria; ^3^Department of Nutritional Sciences and Biobehavioral Health, Pennsylvania State University, University Park, PA, United States; ^4^Department of Food Technology, Safety and Health, Faculty of Bioscience Engineering, Ghent University, Ghent, Belgium; ^5^Department of Epidemiology and Data Science, Amsterdam UMC, Amsterdam Public Health Research Institute, Amsterdam, Netherlands; ^6^The Micronutrient Forum, Washington, DC, United States

**Keywords:** micronutrients, monitoring and evaluation (M&E), effectiveness, logic framework, indicators and metrics, Nigeria, food fortification

## Abstract

Large-scale food fortification (LSFF) programs are widely implemented in low- and middle-income countries (LMIC) to alleviate micronutrient deficiencies. However, these programs may not achieve the desired impact due to poor design or bottlenecks in program implementation. Monitoring and evaluation (M&E) frameworks and a set of agreed indicators can help to benchmark progress and to strengthen the evidence-base of effectiveness in a standardized way. We aimed to formulate recommendations towards core indicators for evaluating the effectiveness of LSFF programs with their associated metrics, methods, and tools (IMMT). For this, we used a multi-method iterative approach, including a mapping review of the literature, semi-structured interviews with international experts, compilation of a generic Theory of Change (ToC) framework for LSFF program delivery, and selection of IMMT for M&E of LSFF programs at key stages along the ToC delivery framework. Lastly, we conducted exploratory, qualitative interviews with key informants in Nigeria to explore experiences and perceptions related to the implementation of LSFF programs in Nigeria’s context, and their opinion towards the proposed set of core IMMT. The literature search resulted in 14 published and 15 grey literature documents, from which we extracted a total of 41 indicators. Based on the available literature and interviews with international experts, we mapped a ToC delivery framework and selected nine core indicators at the output, outcome and impact level for M&E of the effectiveness of LSFF programs. Key informants in Nigeria revealed that the main bottlenecks for implementation of the proposed IMMT are related to the lack of technical capacity, equipment, laboratory infrastructure, and financial resources. In conclusion, we propose a set of nine core indicators for enabling comprehensive M&E of the effectiveness of LSFF programs in LMIC. This proposed set of core indicators can be used for further evaluation, harmonization and integration in national and international protocols for M&E of LSFF programs.

## 1. Introduction

Large-scale food fortification (LSFF), or mass fortification, concerns the addition of one or more micronutrients to industrially processed staple foods or condiments (1, 2). LSFF programs attempt to address micronutrient deficiencies of public health concern, such as deficiencies of iodine, iron, vitamin A, and folic acid, which occur most profoundly in low- and middle-income countries (LMIC). According to the Global Fortification Data Exchange platform^1^ and the Food Fortification Initiative Network,^2^ 126 countries had implemented universal salt iodization programs, 92 countries had mandatory food fortification programs for at least one cereal grain, and 34 countries had mandated the fortification of oil by September 2022, with in addition an unknown number of countries that have implemented fortification of sugar. LSFF is one of the most cost-effective and scalable micronutrient interventions to address inadequacies at the population level (2–6). Nevertheless, LSFF programs may not achieve the desired impact due to poor design and bottlenecks in program implementation. With regard to the latter, for example, almost half of fortified foods in 20 national fortification programs implemented in 12 countries were found not to meet national fortification standards (7). Most LSFF programs, however, do not report any data on compliance, coverage, or impact, leaving major data gaps in monitoring their effectiveness and limiting the ability for corrective measures to be taken (2, 4, 5, 7, 8).

As also highlighted in the Arusha Statement on Food Fortification in 2015,^3^ effective LSFF programs require sound design; to have clear regulatory frameworks; to be continuously monitored for quality assurance (QA) and quality control (QC) procedures at the production site; to have a simple and cost-efficient enforcement system that monitors compliance with fortification standards at production and commercial levels; and to assess equitable coverage and consumption of fortified foods by the targeted population (4, 5). Although an initial generic monitoring and evaluation (M&E) system for food fortification programs was proposed by WHO/FAO in 2006 (1), there is currently no coherent and harmonized M&E framework with a standardized set of essential indicators.

Here, in a multi-method iterative study approach, we aimed to: (1) review published and grey literature for frameworks and commonly used indicators, metrics, methods, and tools (IMMT) to monitor and evaluate LSFF programs; (2) to contextualize the findings from the literature through interviews with international experts; (3) to develop a generic theory of change, followed by a proposed selection of a minimum set of IMMT that are key to track LSFF program effectiveness; and (4) to verify the applicability of this generic theory of change in a local food fortification context, including elucidating the perceptions and barriers towards the proposed set of IMMT by interviewing key informants in Nigeria.

## 2. Methodology

### 2.1. Literature review

To identify relevant sources from the published literature, we used the same search strategy as that used in a previous review by us on M&E indicators for biofortification programs (9). In short, we systematically searched for reviews with detailed descriptions of implementation processes, M&E activities, and IMMT for LSFF programs published between 2010 and 2019. The search was performed in MEDLINE, Cochrane Library, Web of Science, and Scopus (see Supplementary material). Additionally, key documents from the grey literature (e.g., program manuals, workshop reports, and indicator dashboards) were identified through an internet-based search and in the global databases GINA, WHOLIS, and SIGLE. The search was complemented with citation mining (10). In addition, documents provided to us by interviewed experts were added (see Section 2.2). Based on the inclusion and exclusion criteria (Table 1), published reviews and grey literature were selected for further use by two investigators independently (TCA and SRM). Any disagreements between the investigators were resolved through consultation with another researcher from the team (CL). To guide the identification of indicators for M&E from the published and grey literature, we adapted the WHO/CDC logic model for implementing micronutrient interventions in public health (12) to represent the underlying implementation processes of LSFF programs (Supplementary Figure S1). After pilot-testing by CL, two data charting forms were used to extract indicators categorized by the different components of the logic M&E framework (see Box 1 for definitions).

**Table 1:** Inclusion and exclusion criteria for the mapping review.

Inclusion criteria	Exclusion criteria
• Reviews on LSFF programs implemented in low- and middle-income countries^1^ in the last 10 years	• Literature on food fortification programs implemented in high-income countries^1^
• Reviews on large-scale food fortification programs that include staple foods (e.g., sugar, flour, oil) and condiments (e.g., salt, soy sauce, fish sauce, seasoning cubes) that are fortified with one or more micronutrients	• Literature on fortification of targeted foods (e.g., fortified complementary foods for children or pregnant and lactating women or fortified blended foods)
• Documents from grey literature on LSFF, such as program reports, expert consultations, workshop reports, and other relevant documents	• Published literature and grey literature written in languages other than English, Spanish, or French.
• Reviews, other studies, and grey literature written in English, Spanish, or French	• Full-text unavailable
	• Reviews published more than 10 years ago

^1^Low- and middle-income countries and high income countries as defined by the World Bank (11).

BOX 1 Definition of terms.

**Table:** 

Indicator	Quantitative or qualitative factor or variable that provides a simple and reliable means to measure achievement, to reflect the changes connected to an intervention, or to help assess the performance of the intervention.
Metric	System or standards of measurement; a metric refers to the way an indicator is operationalized and expressed in a standardized manner.
Method	A systematic and established procedure for data collection as input for determining metrics and indicators.
Tool	A physical object, device or implement required for data collection.
Input indicator	Financial, human, and material resources used for a program.
Activity indicator	Specific actions taken, or work performed, through which inputs (see above) are mobilized to produce specific outputs.
Output indicator	Products, capital goods, and services that directly result from the activities of a project or intervention, and that are relevant to the achievement of outcomes.
Outcome indicator	Anticipated (or potentially unanticipated) effects of a program in the target population.
Impact indicator	Intermediate-term or long-term outcomes a program in the target population contributes to.
Monitoring	Frequent and continuous collection, analysis, and interpretation of data, and use of the resulting information, on program implementation activities to assess how the program is performing according to predefined criteria and to implement corrective measures.
Evaluation	Systematic and objective assessment of effectiveness and impact of a program on the target population. The aim is to determine the relevance and fulfillment of objectives, quality of performance, outcomes, attribution, cost-effectiveness, and long-term sustainability.

Sources: A2Z Project (21); Allen et al. (1); Oxford Languages (13).

### 2.2. Semi-structured interviews with international experts

We then conducted semi-structured interviews (SSI) with senior international experts on LSFF programs to deepen our understanding of the literature. Based on their track record in the field, nine experts (Table 2) were recruited through purposeful and snowball sampling (14, 15) for an individual (online) interview on a voluntary basis. The purpose of the interview was explained to the participants in detail, both verbally and in writing. SSI participants were asked for verbal informed consent to participate and digitally record the interviews. We aimed to obtain a detailed description of LSFF delivery models, crucial success factors, M&E frameworks, methods, and tools for data collection, and in particular methods for assessing coverage and consumption of fortified foods. SSI guides were developed with guiding questions, including detailed probes on topics of inquiry (16). Data were collected over 5 weeks from March–June, 2021 until data saturation was reached among key themes (16, 17).

**Table 2:** Characteristics of international experts who participated in the SSI.

Organization	Professional role	No
United States Agency for International Development (USAID)	Expert in food fortification	1
East Central and Southern Africa (ECSA) Health Community	Manager	1
World Health Organization (WHO)	Consultant	1
Global Alliance for Improved Nutrition (GAIN)	Nutrition and LSFF expert	1
GroundWork	Co-founder and expert on LSFF	1
Emory University	Professor and senior expert on LSFF	1
Emory University/Food Fortification Initiative (FFI)	Senior Nutrition Scientist and Research Professor	1
Dietary intake experts:		
Harvard T.H. Chan School of Public Health	Expertise in dietary intake assessment methods in LMIC	1
Intake: Center for Dietary Assessment	Expertise in dietary intake assessment methods in LMIC	1

After *verbatim* transcription of the interview recordings by two research assistants, thematic analysis was conducted with the help of the data management software Dedoose version 8.3.47 (18). A codebook with 22 categories of information was developed by SRM and FMG, and cross-checked by SRK. The first coding cycle comprised the application of the initial codes to the transcripts, while the 22 categories were clustered and merged into eight pattern codes (i.e., thematic areas) during a second coding cycle. Further details on the study methodology can be found in Rodas-Moya et al. (9).

### 2.3. Compilation of a theory of change framework and selection of IMMT

Based on the implementation processes of LSFF programs encountered in the literature and as described by international experts, we used a Theory of Change (ToC) approach to create an initial draft framework to show the linkages between the activities, outputs, outcomes, and impact. We then compared this draft to: (1) a generic monitoring and evaluation framework for food fortification programs published by WHO in 2006 (1); (2) the WHO/CDC logic model which we had already used to select IMMT from the literature (Supplementary Figure S1); (3) a generic impact pathway for LSFF developed by GAIN as part of the Fortification Assessment Coverage Tool (FACT) kit (19); and (4) an impact pathway of an LSFF program using multiple food vehicles, i.e., fortifiable foods, from a basket of fortified foods from Costa Rica, which was based on interviews with LSFF experts, program documents, program monitoring data and representative national surveys data (20). The first three of these models represent generic LSFF delivery models constructed with multi-contextual evidence of LSFF programs from LMIC and may thus be transferable across similar settings. The delivery model from Costa Rica represents a rigorously constructed, plausible impact pathway of a fully mature LSFF program that shows the crucial success factors for program effectiveness.

**Figure 1:**
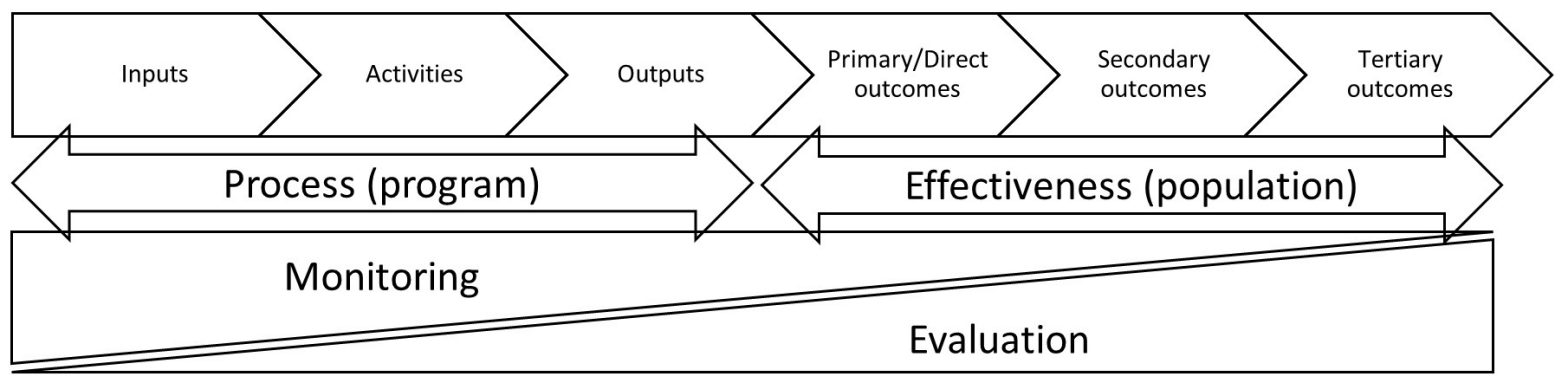
Logic model for M&E of LSFF programs. Based on: A2Z Project, 2008 (21).

We then compiled a final version of the ToC and employed it as an analysis framework for the selection of a set of core IMMT. Since our aim was for the IMMT to measure effectiveness of LSFF programs rather than their design and implementation (Figure 1), we limited our selection to indicators of output and outcome. We aimed to limit the number of indicators to a minimum in order to keep routine M&E practices as simple and cost-effective as possible.

### 2.4. Interviews with key informants on food fortification programs in Nigeria

As a final step, we aimed to verify the applicability of our M&E framework in a local context. We aimed to select an LMIC with mandatory fortification of at least two food vehicles. Nigeria was selected because it fortifies (and biofortifies) multiple food vehicles. Nigeria started its first LSFF program in 1993 with the iodization of salt. This fortification program was successful: the latest national surveys showed that 92% (2018) and 97% (2019) of households had iodized salt available (22, 23). The salt iodization program was followed by mandatory fortification of several other foods in 2000 and beyond, such as vegetable oil (vitamin A), wheat and maize flour (multiple nutrients), sugar (vitamin A), and margarine and butter (vitamin A). However, these programs have been relatively less successful. In a national survey carried out in 2013, only 12.2–33.0% of wheat flour samples were adequately fortified with vitamin A and 1.0–21.0% with iron, while just 14.9–20.2% of vegetable oil was adequately fortified (24). In addition, a sub-national study in the states of Lagos and Kano found that only 5.4–22.7% of households consumed fortified wheat flour and 7.2–7.6% consumed fortified vegetable oil (25, 26).

To reach our aim, we conducted semi-structured, in-depth interviews with key informants. We were specifically interested to hear opinions towards the potential opportunities and barriers when using our proposed IMMT in Nigeria. Participants were identified based on their role in food fortification programs (i.e., being program implementers), either as representatives of government, industry, non-governmental organizations (NGOs), or academia, followed by some snowball sampling as suggested by interviewed participants [(14, 15); Table 3]. The interviews were conducted in person by a team of local data collectors led by FS and AO. Data collectors were trained remotely by SRM and FMG. Interviews were conducted in English and recorded with digital recorders after gaining informed consent from the participants. The recordings were transcribed *verbatim* locally by professional transcribers and cleaned by SRM and FMG for subsequent analysis. Data were collected in January 2022. Participation in interviews was voluntary. All interviewees were informed that they could refuse to participate or withdraw from the study at any point without any consequences for them. Participants received a detailed explanation (both verbal and written) about the purpose of the interview. Data management and analysis followed similar procedures as described for SSI with international experts (see section 2.2).

**Table 3:** General characteristics of SSI participants for LSFF programs in Nigeria.

Sector	Position	No.
Government regulatory agency	Senior officer	1
Private sector (food industry)	Executive	3
Professional	Executive	2
Research institute	Consultant	1
Academia	Academic	1
NGO	Senior officer	3

## 3. Results

### 3.1. M&E frameworks and IMMT extracted from the literature

The literature search resulted in 29 documents: 14 published and 15 gray literature documents. The published literature included 11 reviews (2, 20, 27–35); an effectiveness evaluation study (36); a case study (37); and a review older than 10 years but with extensive information on indicators for M&E of flour fortification programs (38). Nine reviews included information on methods for data collection (20, 28, 30, 33–38); while five reviews (20, 33–35, 38) and two studies (36, 37) included information on tools.

From the grey literature, we retrieved 15 documents, including eight manuals for internal and external quality assurance and control (QA/QC) of fortified wheat flour, salt, sugar, and oil (39–46); a manual that described procedures to enforce the quality of fortified foods at importation sites (47); a manual for commercial inspection of fortified foods (48); a code of practice for micronutrient premix operations (49); a workshop report containing a critical assessment on the root causes of failure in fortification quality; a report of a technical consultation on M&E of LSFF programs (21); and an article on technological aspects of LSFF and public health nutrition (50). Furthermore, the Fortification Assessment Coverage Toolkit (FACT) manual was included to retrieve IMMT for assessing the quality of fortified foods, their coverage, and consumption at population level (19, 51, 52). We did not find any relevant documents containing IMMT for LSFF in the global databases GINA, WHOLIS, and SIGLE.

A total of 16 activity, 11 output, 9 outcome, and 2 impact indicators were extracted from the literature, as presented in Table 4. We did not encounter any specific input indicators in the literature. Activity indicators were centered around the existence of legislation, regulation, and national standards; quality assurance of fortificant premixes, and the presence of internal and external QA/QC processes in support of food fortification. Output indicators concerned the actual production of fortified foods, their market availability, and quantitative verification of adherence to the national standards. Outcome indicators provided information on consumer awareness, coverage and contribution of fortified foods to micronutrient intake (primary/direct outcome). Impact indicators, finally, served to evaluate the effect of a fortification program on nutritional (secondary) and clinical (tertiary) outcomes.

**Table 4:** Indicators (*N* = 41) for M&E of inputs, activities, outputs, outcomes, and impact of large-scale food fortification program, as derived from the published and grey literature.

1. Inputs	2. Activities	3. Outputs	4. Outcomes	5. Impact
Overall resources needed for the program: 1.1. Financial resources 1.2. Human Resources 1.3. Material resources (e.g., equipment/technology)	Regulation and control: 2.1. Legislation, regulation, and national standards for fortification in place^1^ 2.2. Total number of industries that have been licensed to fortify a food vehicle^1^ 2.3. Training plan for food control authorities on the fortification process and sampling to perform the auditing and inspection activities^2^ Micronutrient premix/fortificant at port of entry:^3^ 2.4. Proportion of boxes/containers of premix/fortificant in good order^4^ 2.5. Proportion of premix/fortificant samples with micronutrient content specified in CoA^5^ meeting national standards^2^ Production (internal QA/QC):^6^ Indicators 2.4–2.5 and: 2.6. Number of premix/fortificant containers adequately stored^7^ and according to FIFO^8^ system^9^ 2.7. Amount of food vehicle produced (MT^10^/h or MT/month)^9,11^ 2.8. Amount of premix/fortificant used to fortify the food vehicle (g/min or kg/month)^9^ 2.9. Average quantity of premix/fortificant discharged by feeder (g/min)^9,11,12^ 2.10. Premix/fortificant addition rate^9,11,13^ 2.11. Number of samples (for testing quality) collected every hour with spot density test within target range vs. planned^9^ Enforcement (external QA/QC): 2.12. Proportion of factories with technical audits conducted vs. planned^1^ 2.13. Number of factories with equipment in optimal condition for fortification^1^	Production of fortified food vehicles:^17,19^ 3.1. Proportion of composite samples^14^ with micronutrient indicator/s^15^ meeting national standards (quantitative lab test)^9^ 3.2. Proportion of fortified/unfortified food vehicle produced^1^ 3.3. Tonnage of fortified food vehicle produced^1^ Market availability of fortified food vehicles:^13^ 3.4. Total number of brands of food vehicle^20^ (GAIN, 2019) 3.5. Proportion of brands that are locally produced^20^ Fortification quality of brands^17^ 3.6. Nutrient content of food vehicle brand^20^ 3.7. Proportion of food vehicle brands that are fortified to any extent^20^ 3.8. Proportion of food vehicle brands that are fortified below the minimum of national standard^20^ 3.9. Proportion of food vehicle brands that are fortified according to national standard^20^ 3.10. Proportion of food vehicle brands that are fortified above the national standard^20^ Behavior change communication materials: 3.11. Number of communicational materials distributed and placed or forecasted vs. planned^1^	Consumer awareness of fortified food vehicles:^17^ 4.1. Proportion of consumers aware of nutritional benefits of fortified foods^1^ 4.2. Proportion of consumers aware of the availability of fortified vehicles in the market^1^ 4.3. Proportion of target audience who are aware of key messages promoting consumption of fortified food vehicles^1^ Coverage of food vehicles: 4.4. Proportion of households that consumes a food vehicle (in any form)^20^ 4.5. Proportion of households that consumes a fortifiable food vehicle^20^ 4.6. Proportion of households that consumes a fortified food vehicle^20^ Actual contribution of fortified food vehicles to micronutrient intake: 4.7. Amount of fortifiable food vehicle consumed daily among target groups (e.g., children 6–59 months or women of reproductive age)^20^ 4.8. Actual percentage of daily nutrient requirements met from consumption of a fortified food vehicle among the target population groups^20^ 4.9. Modeled percentage of daily nutrient requirements met from consumption of a fortified food vehicle among the target population groups^20^	Nutritional and clinical outcomes: 5.1. Change in prevalence of a micronutrient deficiency in a target population group^1^ 5.2. Change in prevalence of a specific clinical outcome (e.g., anemia) in a target population group^1^
	2.14. Proportion of factories with GMP^16^ for food safety in place^1,9,11^ 2.15. Proportion of factories with QA/QC procedures for fortification processes^17^ in place^1^ + indicators 2.7–2.11 Behavior change communication: 2.16. Proportion of communicational materials produced^18^ vs. planned^1^			

^1^Peña-Rosas et al. (38); ^2^Makhumala et al. (43–46); ^3^The quality assurance of premix/fortificant at the port of entry is the primary responsibility of export and import agencies; ^4^Packages/boxes/containers should be in good order (i.e., not damaged or broken and hermetically sealed in the case of vitamin A); The integrity of the label (specifying micronutrient levels) should be checked; Lot number, production date, and expiry date should be readable; ^5^Certificate of Analysis; ^6^Quality Assurance/Quality Control; ^7^Stored on top of pallets in a cool, dry place (with air conditioning for vitamin A for oil); ^8^First-In-First-Out system (i.e., first to expire is used first); ^9^Makhumala et al. (39–42); ^10^Metric Ton; ^11^Wirth et al. (37); ^12^This indicator is specific for flour fortification; it should be compared to the target addition rate, see also footnote 13; ^13^ This indicator is specific for flour fortification; assuming a premix target addition rate of 250 g/MT of flour, the addition rate C can be calculated as follows: A = the number of premix boxes used (e.g., 18) multiplied by the weight of each box (e.g., 25 kg), multiplied by 1000 kg, and dividing the resulting quantity by B, the flour production (e.g., 1962 MT), i.e., C = (18 * 25 * 1000)/ 1962 = 229.4 g/MT; hence, in this example the addition rate is 91.6% of the target (C/250); ^14^Composite samples are made up of all samples collected every hour during a shift or a day; ^15^Micronutrient content can be tested with ‘point-of-use’ test-kits; ^16^Good Manufacturing Practices; ^17^This /these indicator/s can be disaggregated by type of food vehicle; ^18^This indicator can be disaggregated by radio or TV spots, posters, counseling sessions, or others; it may also be disaggregated by the target group; ^19^Determined by registering all the available brands in purposively selected marketplaces and retail outlets; ^20^GAIN (19).

### 3.2. Insights shared by international experts

We conducted nine SSI with international experts in total (Table 2). Two relevant primary themes emerged from the SSI dataset for theory building, namely: (1) Feasibility to develop a generic M&E framework for LSFF programs; and (2) Methods to estimate coverage and consumption of fortified foods.

#### 3.2.1. Feasibility to develop a generic M&E framework for LSFF program delivery

When exploring the feasibility of developing a generic M&E framework for LSFF programs for global use, centered around common elements of program implementation, most participants indicated that creating such a framework should be possible under certain conditions. One participant explained:

*“There are many similarities between [fortification] programs across countries and regions. It should be possible to have a generic M&E framework regardless of how the programs are designed. Yet, the instruments for data collection and the questions to feed the M&E systems need to be designed and adapted for specific contexts to get relevant information. Also, the system should be flexible, not prescriptive or rigid to make contextual adaptability possible.”*

                 Academic LSFF expert.

It was broadly acknowledged that any food fortification program cannot happen without participation of the government and support from the industry. Participants mentioned several crucial responsibilities from the side of the government, such as: (1) defining the need and conditions of a fortification program, based on evidence of deficiencies and food consumption patterns of the population; (2) identification of appropriate food vehicle(s); (3) determining the quantities and chemical form of the fortificants to be added and setting national standards (NS); (4) pass legislation; and (5) installing an efficient and reliable enforcement system to ensure compliance with NS. To enable this, designees should be trained that are responsible for enforcement of the legislation by QA/QC at production site and commercial level (e.g., wholesalers, retailers, bakeries). Lastly, governments should level the playing field for industries participating in mandatory fortification program(s). The majority of senior experts stressed that behavior change communication towards consumers does not need to be a priority, since, as stated, when implemented well, the fortified commodities will be consumed anyway.

When exploring the role of the industry in the fortification process, four primary responsibilities were described by participants: (1) Acquiring appropriate technology and inputs (e.g., premix and fortificant) for fortification; (2) Training technical personnel on fortification-related QA/QC processes internally; (3) Mainstreaming fortification QA/QC procedures into existing QA/QC protocols (i.e., not creating new or separate QA/QC programs for fortification) to warrant the production of high quality food products that meet the NS for fortification; and (4) Consistent fortification of the food vehicles at NS as mandated by law, transparent reporting of QA processes and QC results.

#### 3.2.2. Methods to assess the coverage and consumption of fortified foods

Several participants stressed that fortification program effectiveness can only be evaluated when robust data on the quality of fortified foods are available, as well as data on coverage and consumption of fortified foods. Such data allow the estimation of program contribution to micronutrient intake and the extent to which the program contributes to filling nutrient gaps in target population groups. All participants indicated that program evaluation at the outcome level (coverage, consumption, contribution of fortified foods to micronutrient intake, and relief of micronutrient intake inadequacies) should only be carried out by specialists with experience in program evaluation, and only when there is evidence that the program has been implemented as planned and operates efficiently (e.g., the industry is fortifying according to standards). A participant explained:

*“Countries should avoid conducting program evaluations focusing on coverage and consumption of fortified foods and changes in nutritional status attributable to fortification programs because the design of these evaluations is complex. Also, these evaluations should be conducted only for well-established programs, i.e., when fortification is mandatory, the national standards are enacted, enforcement works, and hence, there is evidence that the industry is fortifying according to standards, and coverage is extensive. Once these conditions are met, the effectiveness of the program can be evaluated by an experienced evaluation team.”*

                 Senior nutritionist at an international NGO.

Participants indicated that the evaluation of coverage and consumption of fortified foods should be based on data from representative population samples, for instance surveys with cluster or multistage sampling. Some participants suggested to involve local universities to assess the coverage and consumption of fortified foods to strengthen local capacity in program evaluation.

One participant voiced concern about the proper conduct of surveys to assess coverage and consumption of fortified foods and indicated that survey design could be simplified:

*“…too much complexity has been added to this level of monitoring. Some programs use large surveys with 3,000 households or more and the corresponding collection of samples that subsequently are sent to a laboratory for analysis. This is not needed. Coverage surveys have often a cluster design where data are collected in clusters of, for example, 30 households (= 1 cluster). A sample of the fortified food vehicle can be collected in each of the 30 houses to make a composite sample for the cluster. The composite samples of the clusters are then sent for quantitative analysis to the lab to get the average and standard deviation (variation) of the fortification levels. Those data can be used to estimate the contribution of the fortified food vehicle to micronutrient intake at the household level. This method is also logical because people don’t eat one sample one day, but a collection of samples over a time period. Hence the average of fortification levels collected from a cluster of households can give stronger estimates of average consumption over time than single samples.”*

                 Senior LSFF expert at an international NGO.

The same participant suggested using Household Consumption and Expenditure Surveys (HCES) to estimate coverage and consumption of fortified foods. An advantage of these surveys is that they already work with large sample sizes that permit disaggregation of data at national, regional and sub-regional levels, as well as by household factors to assess equity of coverage. The participant explained:

*“HCES uses large sample sizes, for example, 24,000 households are surveyed, and data can be disaggregated by region, type of residence (urban/rural), and economic quintile. These surveys have a food module that contains a list of more than 100 foods consumed at the household with reported weights [.....]. Purchase and sometimes consumption of the foods on the list are reported over seven or 15 days. It is also possible to check which of these foods are […] fortifiable or already fortified foods. HCES data allow to estimate consumption of foods at the household level using the average (fe)male equivalent method* ^4^ *.”*

                 Senior LSFF expert at an international NGO.

We further enquired whether 24-h recalls (24hR) or Food Frequency Questionnaires (FFQ) should be used to provide estimates of coverage and consumption of fortified foods, rather than those calculated based on average (fe)male equivalents with HCES data. The majority of participants indicated that 24hR can provide more accurate estimates of food intake than HCES. However, all participants acknowledged that conducting 24hR in most LMIC program settings will be challenging, and that there is a need to simplify methods. A participant explained:

*“Consumption assessment is the science of approximation. 24hR are also an approximation. Why do we want to be so precise when we are talking about program evaluation at the population level? A scientific mind could be simple or complicated. Some researchers want to do things so strictly that they become impractical. HCES gives you highly relevant data for programs because it allows you to see what segments of the population are covered and what aren’t. This has programmatic value because it helps crafting strategies to target uncovered population groups. You monitor and evaluate a program because you want to steer it to achieve its goals. Program evaluation is not an epidemiological study”*

                 Senior LSFF expert at an international NGO.

All participants also indicated that the integration of a module to assess coverage and consumption of fortified foods in large cross-sectional surveys, such as Demographic and Healthy Surveys (DHS) or the UNICEF Multiple Indicator Cluster Survey (MICS), could be a good alternative for establishing a separate routine evaluation system for LSFF. When asking participants about the time frame for assessing coverage and consumption of fortified and fortifiable foods, all of them indicated that once in 5 years would be sufficient.

Finally, we explored the possibilities of using existing diet quality tools, such as the Minimum Dietary Diversity for women (MDD-W) (54) and the Global Diet Quality Score (GDQS) (55, 56), to monitor the coverage of LSFF programs. However, international experts unanimously discouraged the use of these tools for M&E of LSFF programs, because they would provide insufficient actionable information.

### 3.3. Compiling a generic ToC framework with selected IMMT for monitoring effectiveness of LSFF programs

**Figure 2:**
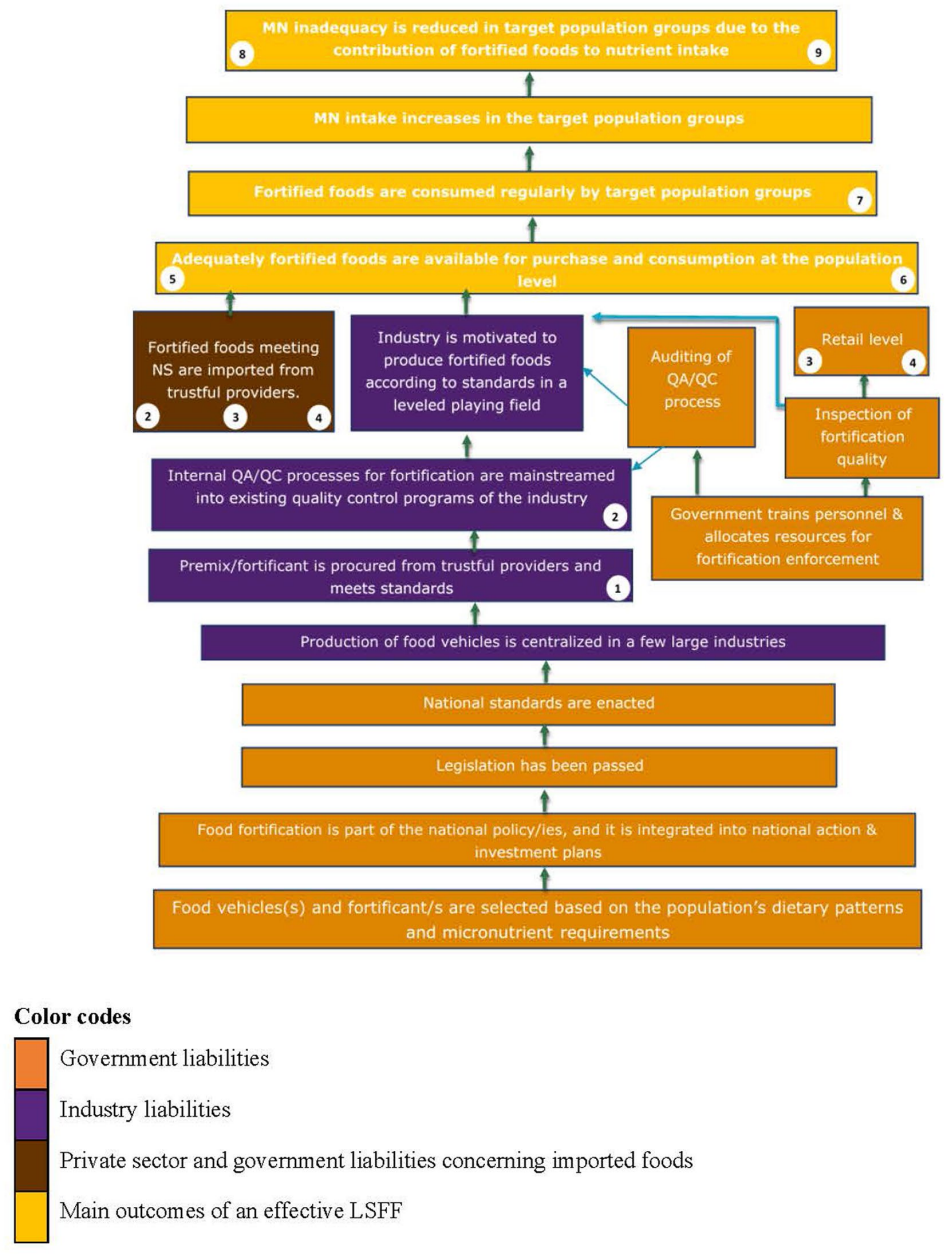
Generic theory of change for large-scale food fortification programs. The numbers in white circles correspond to the indicators shown in Table 5.

The final version of the ToC framework can be found in Figure 2. Out of the indicators identified through the mapping review (Table 4), a set of nine core indicators (Table 5) was selected such that the most critical implementation stages of LSFF are covered, as indicated in Figure 2. The selection includes one indicator at the activity level (indicator #2.5: Proportion of premix/fortificant samples with micronutrient content specified in CoA meeting national standards), because it was deemed crucial by most of the international experts to verify that the LSFF program is properly implemented before performing an effectiveness evaluation.

**Table 5:** Proposed set of high-level indicators, metrics methods, and tools for M&E of large-scale food fortification programs.

Indicator and metric	Methods for data collection	Tools
Output level		
1. Proportion of premix or fortificant samples with micronutrient content specified in CoA^a^ meeting NS^b^$\frac{\left(\;\begin{array}{c} \text{Nr}\;\text{of lots of premix with micronutrient}\\ \text{content specified in}\;\text{CoA}\;\text{meeting}\;\text{NS}\;\; \end{array}\;\right)}{\left(\text{Total}\;\text{nr}\;\text{of lots of premix}\right)}*100$	This indicator is constructed with data from laboratory analysis for the quantitative determination of micronutrient content in samples. The laboratory analysis results must match the values specified in the CoA and premix label and comply with NS.	NS, Laboratory reports, CoA, and product labels.
2. Proportion of monthly composite samples with micronutrient indicator/s meeting NS^c^$\frac{\left(\;\begin{array}{c} \text{Nr}\;\text{of monthly composite samples with}\;\\ \text{micronutrient content meeting}\;\text{NS}\; \end{array}\;\right)}{\left(\text{Total}\;\text{nr}\;\text{of composite samples}\right)}*100$	This indicator can test the quality of imported foods at port of entry and the quality of locally produced foods at the production site by the industry and by enforcement authorities. At the port of entry, three samples of 500 g or 100 mL each are collected from each brand and consignment that has been accepted in the country. The samples are collected in 5000 g or 1000 mL containers labelled with the name of the imported brand and the sampling date. The containers are well-closed and kept in a dark, dry, and cool place. Accepted samples of the same brand that arrive in subsequent consignments are combined by adding them to the 5000 g/1000 mL container for that brand. The corresponding date of each and every consignment needs to be added to the composite sample. Once the container is full (3 consignments), it is closed and stored. New containers should be used for additional samples. Once a month, the composite samples are sent to a reference laboratory to test compliance with NS. At the production site, composite samples should be routinely made daily, as part of the QC process. The composite samples of fortified foods are made by collecting samples of 500 g of food vehicle (200 mL for oil) per hour, e.g., during an 8-h shift. The samples are then mixed to make a composite sample (e.g., a combined sample made with eight samples of 500 g each). Once or twice a week, the industry can take a few of these composite samples (e.g., 3–5) and send them to the laboratory to test compliance with NS. Eighty percent of the composite samples should comply with NS with the average close to the specified factory addition level of the micronutrient/s for a specific food vehicle. During enforcement inspection, the food control authority checks that “daily composite samples” for the last 30 working days are adequately stored. The inspector randomly chooses three daily composite samples from the last month’s production, writes down the production date, estimated micronutrient level, and any other information labelled in the sample identification, and sends them to a laboratory for quantitative analysis to verify compliance with NS.	Manual for Inspection of Fortified Foods at Importation Sites, manuals for internal and external QA/QC for a specific food vehicle (available at: http://a2zproject.org/node/74), containers for storing the samples, labels, inspection forms, and laboratory reports.
Outcome level		
3. Proportion of food vehicle^d^ brands that are fortified to any extent^e^$\frac{\left(\;\begin{array}{c} \text{Nr}\;\text{of food vehicle brands that}\;\text{are}\;\\ \text{fortified to}\;\text{any}\;\text{extent}\; \end{array}\;\right)}{\left(\text{Total}\;\text{nr}\;\text{of food vehicle brands}\right)}*100$	These indicators can be constructed with data from a Market Assessment, as described by the FACT. The Market Assessment Survey is based on selecting a target group geographically circumscribed. A purposive multi-stage sampling strategy with different levels of markets (e.g., wholesalers, supermarkets, and retail stores) may be used. For constructing these indicators, 5–10 samples from different production batches (or different sizes of packages) across all market hubs should be collected for each brand of fortified food vehicle registered. The samples are then sent to a laboratory for quantitative analysis of micronutrient indicator/s. The FACT indicates that the quantity of food required for the sample differs depending on the food vehicle type. For example, for oil samples, 300 mL and needed, while for salt, 50 g is recommended. To design and conduct a Market Survey, it is advised to integrate an experienced team in survey design and train the data collectors before conducting the survey. Alternatively, these indicators can be constructed with data from inspection of fortification quality at retail, wholesale, and bakeries. The sampling strategy is convenient-based and focuses on salt, sugar, flour, and oil. This monitoring allows for the detection in the market of brands that are not approved by the Ministry of Health or do not comply with national standards. It also helps to confirm whether brands previously inspected in factories and importation sites fulfill the requirements claimed by inspectors during the external monitoring process. Samples of ~0.5 kg or 0.1 L of each brand of each fortified food are collected in the whole seller, retailer, or bakery. If the food is not available in such quantities, sufficient packages should be collected to make up the specified weight, e.g., two packets of 250 g. The samples are packed into a box and taken to the Food Control office, split in half, and sent the other half labelled adequately to the laboratory for subsequent qualitative and quantitative testing. For quantitative testing, composite samples of up to 5 different samples per brand are assembled to assess the average micronutrient content.	The FACT kit and associated tools (available at: https://www.gainhealth.org/resources/reports-and-publications/fortification-assessment-coverage-toolkit-fact) Manual for Commercial Inspection of Fortified Foods (available at: http://a2zproject.org/node/74), Inspection forms, Boxes to pack the samples
4. Nutrient content of food vehicle brand^e^ Metric depends on the type of nutrient and NS requirement		
5. Proportion of households that consume a fortifiable food vehicle $\frac{\left(\;\begin{array}{c} \text{Nr}\;\text{of households where the fortifiable}\;\\ \text{food vehicle was consumed}\; \end{array}\;\right)}{\left(\text{Total}\;\text{nr}\;\text{of households}\right)}*100$	These indicators can be constructed with data from a Household Assessment described by the FACT kit. The household assessment is based on selecting a target group (most frequently children 6–59 mo. and women of reproductive age) geographically circumscribed. A multi-stage sampling design may be used. The sampling strategy should make it possible to distinguish between households from rural areas, households at risk of acute poverty, households with low socioeconomic status, households at risk of food insecurity, households with women of reproductive age not meeting minimum dietary diversity, and households with poor infant and young child feeding practices. Alternatively, the FRAT recommends a cluster sampling design. Clusters are small administrative units, such as villages in the rural setting or wards in the urban setting. The survey will select 30 clusters from each sampling area (this allows for a reliable and representative estimate to be made for a population group while minimizing logistical requirements). The Cluster Survey can also be piggybacked on existing representative surveys such as the UNICEF Multiple Indicator Cluster Survey (MICS) or Demographic and Health Surveys (DHS).	The FACT kit and associated tools and the Fieldwork Manual for the Household Assessment (available at: https://www.gainhealth.org/resources/reports-and-publications/fortification-assessment-coverage-toolkit-fact). The FRAT tool and its survey forms are available at (https://www.nutritionintl.org/wp-content/uploads/2017/07/FRATguidelines2003_Nov_2008.pdf)
6. Proportion of households that consumes a fortified food vehicle $\frac{\left(\;\begin{array}{c} \text{Nr}\;\text{of households where}\;a\;\text{fortified}\;\\ \text{food vehicle was consumed}\; \end{array}\;\right)}{\left(\text{Total}\;\text{nr}\;\text{of households}\right)}*100$		
Impact level		
7. Estimated intake of “fortifiable” food vehicles per adult (fe)male equivalent at households Grams of food vehicle consumed per day by the target group	This indicator can be constructed from Household Consumption and Expenditure Surveys (HCES) to estimate apparent food consumption, including consumption of fortified foods, based on the adult (fe)male equivalent formula proposed by the Food and Agriculture Organization.	See: Weissel and Dop (53).
8. Reduction in the proportion of the target population at risk of micronutrient inadequacy due to intake of fortified foods $\frac{\left(\begin{array}{l} \text{Percentage}\;\text{at}\;\text{risk of micronutrient}\\ \text{inadequacy after food fortification} \end{array}\right)}{\left(\begin{array}{l} \text{Percentage}\;\text{at}\;\text{risk of micronutrient}\\ \text{inadequacy before food ortification} \end{array}\right)}*100$	Indicators 8 and 9 can be constructed by combining consumption data from the Household Assessment, or Cluster Surveys, or HCES, based on adult (fe)male equivalents, with fortification quality data generated by indicator 4. A highly skilled team with rich experience in program evaluation and statistical analysis skills is required to construct indicators 7–9.	Proportion of the population at risk of micronutrient adequacy can be calculated as the percentage of the population with nutrient intake below the Estimated Average Requirement (EAR cut-point method), or as the percentage of the population with dietary nutrient density below critical nutrient density thresholds. See: Tang et al. (57)^f^
9. Proportion of population per stratum that remains at risk of micronutrient inadequacy in spite of consuming fortified foods $\frac{\left(\begin{array}{l} \;\text{Nr}\;\text{of people}\;\text{per}\;\text{stratum that remains}\;\\ \text{at}\;\text{risk of micronutrient inadequcy}\; \end{array}\right)}{\left(\text{Total}\;\text{nr}\;\text{of people}\;\text{per}\;\text{stratum}\right)}*100$		

^a^Certificate of analysis. ^b^National standards. ^c^ This indicator can be used to test the quality of imported foods at port of entry and to test the quality of foods at production site. ^d^Food vehicle refers to a fortifiable food. ^e^The nutrient content of composite samples per brand can be compared to the NS ranges to assess compliance. ^f^Note that neither of these methods can be used to evaluate the risk of inadequacy of iron intake for women of reproductive age, because their requirement distribution is skewed to the right due to menstrual iron loss. For this population group, the Probability of Adequacy approach can be used instead.

Indicators #1 and #2 were extracted from the manuals prepared by the East, Central and Southern Africa Health Community (ECSA-HC) with support from partners in the Regional Food Fortification Initiative (39–48). These modules have been used already for long to monitor the quality of premix and fortified foods. Indicator #1 is used to verify that the premix complies with NS. It can be used by the industry to check the quality of premix before fortifying the food vehicles of interest (39–42). Indicator #2 tests whether the fortified food vehicles comply with the NS based on the random selection of 3–5 composite samples within a given month, to be repeated 1–2 times per year. It can be used to test the average content of micronutrient indicator/s of imported foods (per brand) at the site of import (47), and of locally produced foods (per brand) at the site of production by the industry (39–42) as well as by enforcement authorities (43–46). Monitoring compliance of premix and fortified foods with NS is crucial for ensuring that the fortified foods have the expected quality (1, 58).

Indicators #3–6 were extracted from the FACT (19, 51, 52). The FACT is a comprehensive toolkit designed to undertake thorough evaluations of LSFF programs. It assesses the availability of fortified foods and their quality at the market and/or household level; coverage, consumption, and the contribution of fortified staples and condiments (and targeted foods to specific population subgroups) to micronutrient intake (52). We only extracted those indicators from the FACT that assess the availability and quality of fortified foods at the market level (indicators #3 and #4, respectively), the availability of fortifiable food vehicles (i.e., centrally produced by the industry and thus amenable for fortification), and the consumption of fortified foods at the household level (indicators #5 and #6, respectively). The latter indicators are particularly useful to assess the actual reach of the LSFF program, and the potential reach and impact of the program if all the fortifiable food vehicles would be fortified. Hence, these indicators are crucial for decision-making on whether scaling fortification of specific food vehicles could help to close nutrient gaps at the population level.

Indicators #7–9 were recommended by a senior expert on LSFF who participated in the SSI. These indicators estimate the consumption of fortified foods (indicator #7), the reduction in the proportion of the target population at risk of micronutrient inadequacy due to the consumption of fortified foods (indicator #8), and the proportion of the population per strata of interest that remains at risk of micronutrient inadequacy despite consuming fortified foods (indicator #9). These indicators can be constructed with a mathematical modelling framework using HCES data and the FAO adult (fe)male equivalent formula to estimate apparent food consumption at the household level (53, 59). This methodology has already been applied to evaluate the contribution of fortified foods to micronutrient intake of women of reproductive age in Malawi (57), although some caution should be exercised when using HCES data for LSFF program design (60). HCES data can be used to model the potential contribution of fortified food vehicles, e.g., oil, flour, or sugar, to closing micronutrient gaps in the general population as well as in vulnerable population subgroups. Different fortification scenarios can be modeled, for instance, the contribution of a single fortified food vehicle vs. the combination of two or more food vehicles for reducing micronutrient inadequacies in the target groups, which elicits crucial information for program decision-making (57). Although conducting this kind of assessment also requires technical expertise, the analysis can be performed with secondary data sources, which simplifies the evaluation process and reduces costs.

### 3.4. Exploratory study on the delivery of LSFF programs and their M&E frameworks in Nigeria

Four primary themes, aligned with the study aims, emerged from the 11 key informant interviews in Nigeria for theory building, namely: (1) Delivery models of LSFF programs in Nigeria; (2) Perceived barriers to the implementation and M&E of LSFF programs; (3) Perceived opportunities to improve M&E of LSFF programs at the outcome and impact level; and (4) Perceptions toward the proposed set of core IMMT (Table 5).

#### 3.4.1. Delivery framework of LSFF programs in Nigeria

**Figure 3:**
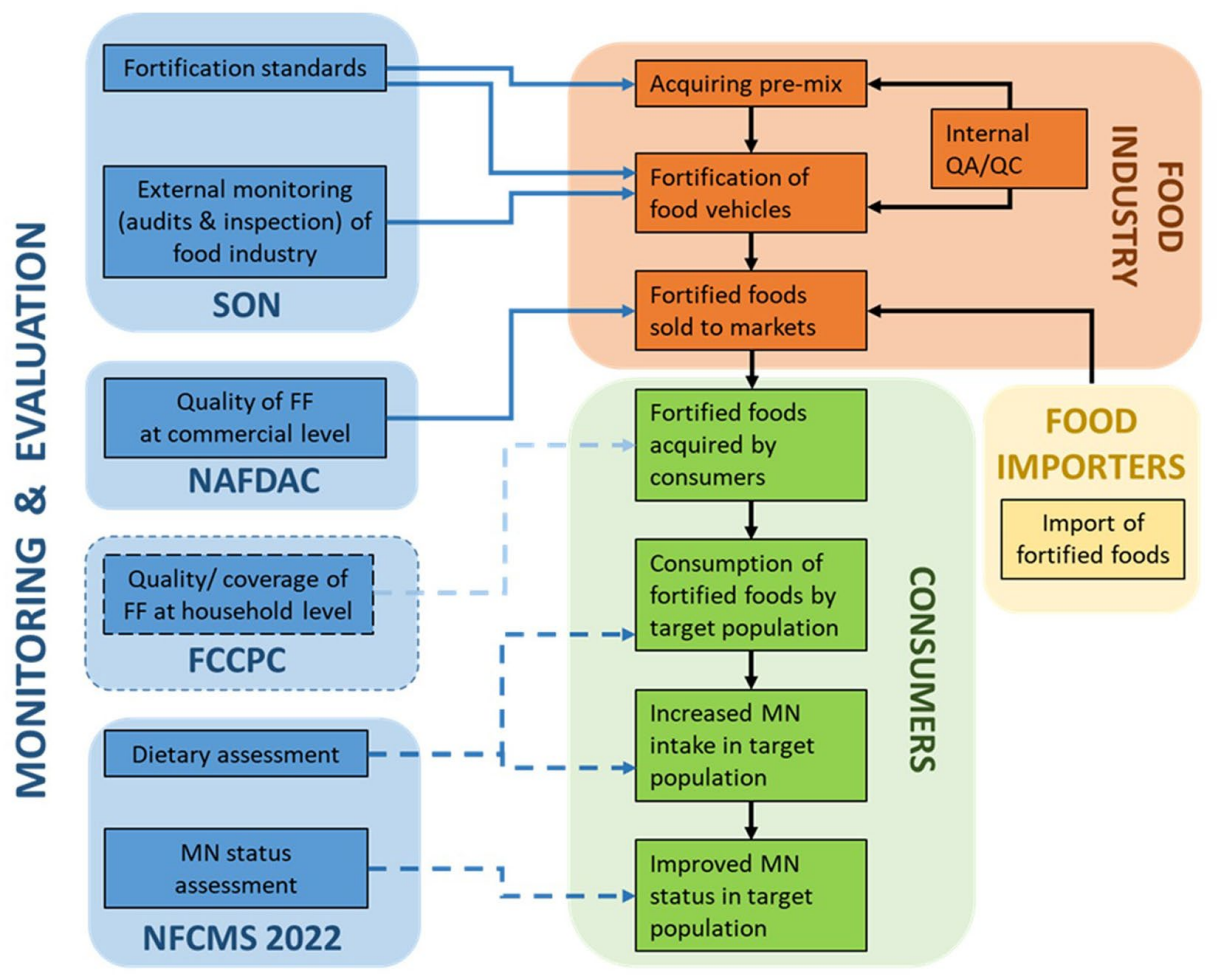
Theory of change (ToC) framework for LSFF program delivery in Nigeria, as emerged from interviews with local key informants. The solid blue lines represent M&E actions that are currently taking place, while the blue dotted lines represent M&E actions which are supposed to take place but were described by key informants as uncertain or not being carried out. SON, standards organization of Nigeria; FF, fortified foods; NAFDAC, National Agency for Food and Drug Administration and Control; FCCPC, Federal Competition and Consumer Protection Commission; MNFCMS, Micronutrient National Food Consumption and Micronutrient Survey; QA/QC, Quality assurance and control.

Nigeria’s LSFF programs are firmly grounded in the broader spectrum of its nutrition policies, with a clear legislative framework. LSFF is implemented as a multi-sectoral effort between government and industries, with support from international NGOs. Two governmental institutes are put in place to enact LSFF programming and to enforce implementation: the Standards Organization of Nigeria (SON), whose mandate is to set and review the industrial standards for fortified foods, as well as monitor compliance at industry level; and the National Agency for Food and Drug Administration and Control (NAFDAC), which is responsible for Good Manufacturing Practices in general, and is expected to monitor food fortification levels at the commercial level (i.e., retail, wholesale, and bakeries). Coordination of the LSFF programs is organized through various structures. The National Advisory Council on Micronutrient deficiency control (or: MNDC Taskforce) is tasked with the coordination of all micronutrient interventions in general, including LSFF. Salt testing for iodine content is specifically coordinated between the Federal Ministry of Health and the Federal Ministry of Education. The Universal Salt Iodization/Iodine Deficiency Disorders Task force is a multistakeholder platform with its secretariat at SON, and coordinates activities specifically to control iodine deficiency. The National Fortification Alliance (NFA), with its secretariat at NAFDAC, is another multistakeholder platform that fosters (mandatory) food fortification, and represents regulators, food producers, and NGOs involved in food fortification. One of our key informants explained that the NFA is the main stakeholder that facilitates SON to conduct audits and inspections in the factories, and NAFDAC to monitor fortification at the commercial level. The NFA also monitors the periodic reporting by SON and NAFDAC. We summarized the descriptions of the delivery of LSFF programs by SSI participants in a ToC framework specifically for Nigeria (Figure 3).

#### 3.4.2. Perceived barriers to the implementation and M&E of LSFF programs in Nigeria

Limited financial and human resources, as well as lack of adequate training for monitoring staff and limited adequate facilities, equipment and consumables to enable staff to perform their duties, were seen as important barriers to M&E of LSFF in Nigeria. Another important barrier mentioned by most interviewed stakeholders was the long-standing trust issues between the monitoring agencies and food manufacturers. A general lack of structure, harmonization, and coordination between stakeholders in monitoring activities was also mentioned, for example resulting in different compliance rates being reported for the same food vehicle. The current M&E system, which consists of deriving coverage data from sporadic spot checks only, both at the industry- and at the market-levels, was perceived to provide unreliable information on fortification quality and coverage of LSFF. The government stakeholders explained their reluctance to take on a more complex approach to monitor product quality because of the costs and the limited capacity available.

Also, the difficulty to monitor the informal market of some fortifiable food vehicles (e.g., sugar and vegetable oil, often sold in small, unmarked containers in informal markets) was mentioned to be a barrier. The participants explained that the informal market makes up a significant portion of the total market and is not regulated, which the larger food manufacturers perceive as an unfair playing field.

*“They [the government regulatory agencies] know, so that is part of the inefficiencies, so we see a banned product, that majority of it may not be fortified, being sold everywhere. But the issue is - I stay in my factory every time, and they [SON] come to check on me, and I’ve been wondering what they are doing to the ones that are contraband, or the ones that are informally done and not being fortified.”*

                 Executive from the Nigerian food industry

Further, it was mentioned that there is a lack of oversight over the regulatory agencies, which may allow poor practices and/or corruption to occur. Additionally, government officers in charge of monitoring may be reluctant to take actions or report instances of non-compliance because retaliatory episodes of violence have happened in the past (e.g., to a SON agent).

*“Now if you have a regulatory officer in that situation that finds somebody that has contravened, maybe it’s a NAFDAC official that went to the market and discovered that thing, and he knows that if he says anything his life is at stake, his office is a public office, anybody can come to the office for revenge […]”*

                 Senior Nigerian government officer

Limited infrastructure and technical capacity to collect and analyze data in Nigeria was also mentioned to be a barrier to effective monitoring:

*“[…] the 2021 National Food Consumption and Micronutrient Intake Survey, and part of the issue that came up is that we are exporting the food samples and the blood samples that we are collecting, because we don’t have adequate labs to monitor the level of the micronutrients. So, to do a micronutrient survey now, we can’t do it locally. It can’t be done locally, because we can’t assess the level in the sample. Some samples are going to China, some are going to maybe Germany, and I am not sure - maybe some are going to US or South Africa. They are sending samples in different directions, because we don’t have the right infrastructure locally. So, all of this monitoring these things, there is no point collecting samples if there is no one that would […] analyze the samples and say, this is the level of what we are looking for, in the sample. So it is impossible to have an M&E framework without that. […] If we are going to have an effective monitoring system, it has to be affordable. If you are exporting your samples for testing, there is no way your M&E system is affordable.”*

                 Academic.

#### 3.4.3. Perceived opportunities to improve M&E of LSFF programs at the outcome and impact levels

Key informants mentioned two mechanisms that can be harnessed for M&E of LSFF programs at the outcome and impact levels. First, the “diet questionnaire” in the National Food Consumption and Micronutrient Survey conducted in 2021 (Federal Government of Nigeria/IITA, 2022), can be used to assess the consumption of fortifiable and fortified foods. According to some of the key informants, at the time of the interview the NFCMS was still finalizing the optimal way of interpreting the results related to fortification and deciding on the best approach to analyze the collected data. The quote below illustrates some of the challenges faced in interpreting the survey results when assessing fortified food intake:

*“Are you going to base it on the national standard? That’s the assumption that it is fortified adequately. Or what do you want to base that on? What’s going to be your denominator? And even the numerator? So that one is still a puzzle that we cannot solve. […] But if it is food frequency, you do this: is it fortified, yes or no? Then if you collect samples, fine! You follow up with derived… okay this thing is fortified, if the person mentions the brand, look at fortification status of the product it is adequately fortified, you can say that. If it is not, you know if it’s to an extent, to what extent? One-quarter, two and half, three-quarter; it will complicate the whole thing. […] We are having that issue; I say where we have multiple vehicles with different status of fortification, how are we going to manage that? […] So how do you want to manage within the recipe?*

                 Senior government officer

The second mechanism mentioned by some key informants is the periodic impact assessment of the salt iodization program conducted by UNICEF, where subsets of school children are asked to bring salt samples from home to school (cluster sampling design), which are then tested for iodine content.

#### 3.4.4. Perception toward the proposed set of core IMMT

When exploring perceptions, barriers, and enablers for using the indicators as proposed in section 4, most Nigerian key informants found it quite challenging to give their feedback. They focused on identifying barriers, rather than opportunities. Table 6 summarizes the most salient perceptions that emerged for indicators deemed most relevant and applicable to the Nigerian context by the participants.

**Table 6:** Summary of perceptions toward the high-level indicators for Large-scale Food Fortification programs.

Indicator definition	General perception	Barrier	Description
1. Proportion of premix or fortificant samples with micronutrient content specified in CoA meeting NS^a^	–	–	–
2. Proportion of monthly composite samples with micronutrient indicator/s meeting NS	This indicator was generally perceived to be important. The widespread hesitation among participants was limited to the use of the term “monthly”: the time frame was not seen as important, but something that should be flexible and adapted to the specific circumstances.	It is resource-intensive to collect data to track this indicator in terms of human resources, equipment and financial resources.	The challenges associated with the data collection necessary to track this indicator are: training, human resources, laboratory equipment and consumables in order to test the samples.
3. Proportion of food vehicle brands that are fortified to any extent	There is disagreement with regard to this indicator: some participants saw it as useful; others raised the issue of the wording “to any extent,” which can be seen as a free pass for inadequate fortification levels.	Fortification “to any extent” can be seen as being sufficient by the food industry; so this indicator may be misleading. It is also resource intensive to collect data for this indicator.	Allowing this indicator to be known by the private sector may convey the misleading message that any amount of fortification is accepted, as opposed to aiming at achieving adequate fortification levels as required by national standards.
4. Nutrient content of food vehicle brand	There was uncertainty among all participants about the definition, usefulness, and applicability of this indicator to M&E of LSFF.	Adequate technical capacity and financial resources are needed to use this indicator, which can be a limiting factor in this context.	There was a perceived need to undertake a market assessment for this indicator. The cost of collecting the data with a market assessment can be a limiting factor for Nigeria; the logistics needed were also perceived to be a potential challenge.
5. Proportion of households that consume a fortifiable food vehicle	–	–	–
6. Proportion of households that consumes a fortified food vehicle	This indicator is perceived as important, but very challenging to track and to collect data for it. It is perceived as a resource-intensive and time-consuming indicator to monitor, thus only seen as a 5-year exercise, rather than a regular monitoring activity.	Consumers may not be aware or remember whether the food they ate was fortified or what brand it was.	This indicator was perceived as a resource-intensive and time-consuming indicator to monitor.
7. Estimated intake of “fortifiable” food vehicles per adult female equivalent at households	–	–	–
8. Proportion of the target population that reduces micronutrient inadequacy because intake of fortified foods	This indicator is perceived as important, but at the same time very challenging and expensive to track. Most participants thought that data for this indicator could be collected every 10 years.	Expensive information to collect, and difficult to attribute changes in micronutrient status to fortified food intake.	The methods required to collect the data to track this indicator are very costly (e.g., dietary intake assessment), micronutrient status in the nationally representative survey, and as such can only be collected every decade (at best).
9. Proportion of population per stratum that remains with micronutrient inadequacy in spite of consuming fortified foods	–	–	–

^a^National standards.

Indicators #2 (‘Proportion of monthly composite samples with micronutrient indicators meeting NS’), #6 (‘Proportion of households that consumes a fortified food vehicle’), and #8 (‘Reduction in the proportion of the target population at risk of micronutrient inadequacy due to fortified foods’) were all perceived as important; yet, important barriers mentioned were the required resources in terms of technical capacity, infrastructure, equipment, and finances (indicators #2 and #8), as well as the difficulty for consumers to know or remember if they consumed fortified foods (#6). In addition, the time-frame for monitoring of these three indicators was discussed, where our key informants felt that indicator #2 should not be limited by the word ‘monthly’, while for indicators #6 and #8 a timeframe of 5 and 10 years, respectively, was seen as appropriate.

Respondents were uncertain about the definition, usefulness, and applicability of indicators #3 (‘Proportion of food vehicle brands that are fortified to any extent’) and #4 (‘Nutrient content of food vehicle brand’). Specifically with regard to indicator #3, our key informants raised that the wording ‘to any extent’ could provide manufacturers with a free pass for inadequate fortification. For both indicators, it was mentioned that available resources would be a limitation for data collection.

We did not receive any clear feedback on indicators #1 (‘Proportion of premix or fortificant samples with micronutrient content specified in CoA meeting NS’), #5 (‘Proportion of households that consume a fortifiable food vehicle’), #7 (‘Estimated intake of “fortifiable” food vehicles per adult (fe)male equivalent at households’) or #9 (‘proportion of population per stratum that remains at risk of micronutrient inadequacy in spite of consuming fortified foods’).

## 4. Actionable recommendations

Monitoring of output indicators (indicator #1–4) requires standardized food sampling and measurement procedures. As per the exploratory findings from Nigeria, there is need for support to build local M&E capacity in some LMIC. This may entail the development (or strengthening) of technical capacity for data collection, processing, storage, and reporting; as well as the development (or strengthening) of laboratory capacity, including training of skilled personnel, ensuring good laboratory practices, and the availability of adequate equipment and inputs for food sampling and analysis of micronutrient content. Capacity building and strengthening local entities will facilitate the implementation and continuity of the proposed M&E framework, while disconnecting the system from its reliance on external technical capacities and donor funding.

As also shown here for Nigeria, building trust between government and industry is a crucial factor for the successful implementation of LSFF programs. Cooperation of the industry can be triggered by full involvement in all aspects of LSFF programming and ownership, rather than just providing a regulatory framework and enforcement. Activity and output indicators (indicators #1–4) require standardized sampling and measurement procedures, but also transparent reporting as a means to improve efforts where needed. The introduction of the Micronutrient Fortification Index (MFI)^5^ in Nigeria and other countries, pioneered by Technoserve, may bring positive change in industry compliance. At the same time, enforcement by the government will still be required to guarantee compliance and to level the playing field.

Concerning indicator #2, based on the exploratory interviews with local stakeholders in Nigeria, it should be clarified that ‘monthly’ means ‘in a given month’, rather than ‘every month’. Whereas factories are supposed to continuously monitor their own fortification performance by weekly sending out 3–5 composite samples (i.e., composed of samples collected hourly during an 8-h shift) to a laboratory, the enforcement authority is expected to pay an unannounced inspection visit once or twice per year to cross-check compliance. During such a visit, three randomly chosen composite samples may be selected from the last month’s production to be send to a certified laboratory. The average micronutrient content of the composite samples will be taken as approximation of the micronutrient content in a fortified food vehicle at that particular production site and be compared to NS. Regarding the suggestions of the local key informants to rephrase indicator #3 by replacing the words ‘fortified to any extent’ with ‘adequately fortified,’ we would like to emphasize that this indicator is meant to track coverage at the commercial level, and not to monitor compliance with NS at the factory level. Lastly, the concern of respondents related to indicator #6 that consumers may not know whether they are consuming fortified foods: this indicator is constructed based on information on consumption of food vehicle brands; hence, consumers do not need to reveal any direct information on fortification because this will be captured by indicator #3 and #4.

For monitoring of outcome and impact indicators (indicators #5–9), information on dietary intake is required. We encountered divergent views on the use of 24hR methods versus FFQs to capture the contribution of fortified foods to the daily dietary intake of consumers. Conducting 24hR is laborious and requires advanced technical expertise, while FFQ’s are easier to administer but require rigorous design and thorough validation before they can be used for a specific purpose. Although conducting 24hR at national scale has its challenges, they provide good quality data and are an important component of periodic nutrition and dietary surveillance systems. If such a surveillance system is in place, it can also be used as a design and monitoring tool for LSFF programs, as shown by a case study in Cameroon (61–63). In the future, collection of 24hR and post-collection data processing may be simplified by technological solutions (64, 65). However, consumption data at the household level, such as collected for HCES, provides sufficient and more cost-efficient information to monitor nutrient intake from fortified foods at the (adult) population level within an acceptable error margin, while allowing for subgroup analyses, and may therefore be preferred (59, 63, 64).

Periodic surveys (e.g., with intervals of 5 years) can ensure regular and sustainable data collection on the coverage and consumption of fortified foods, and their contribution to diet quality. For mature programs with high coverage, integration of indicators #5–9 into modules of existing representative national data collection systems, such as National Nutrition and Health Surveys, HCES, or DHS, will be the most cost-efficient means for routine M&E of LSFF programs. Such surveys are already implemented in many LMIC with typical update frequencies of once per 3–10 years, which was regarded as sufficient as indicated by the experts and the local key informants. Further work is required to determine the feasibility of incorporating data collection for the recommended indicators into such existing surveillance systems. When larger-scale national surveys are not available or when a new LSFF program is introduced, sentinel site surveys can also be useful. Sentinel site-based monitoring systems can generate timely actionable information to improve program implementation at a relatively low cost with proven effectiveness (36, 38, 66). The government of Costa Rica, for example, uses such surveys in strategic geographic circumscriptions to track changes in the prevalence of iron deficiency and anemia attributable to their LSFF programs (36). As reflected by the selected indicators, it was not deemed required to assess biochemical micronutrient status based on blood samples for M&E of LSFF programs. Collection of such data is costly and unlikely to be specific for the impact of food fortification. Nevertheless, monitoring of micronutrient deficiencies at the population level is an important component of periodic dietary and nutrition surveillance in general.

Previously, we have compiled a ToC framework with IMMT for biofortification programs, using very similar methodology as described here (9). Initially, we aimed to develop one ToC framework that would capture both biofortification and LSFF programs. However, early on in the process we discovered this to be cumbersome, because of the distinct differences in the implementation pathways between biofortification and LSFF programs. This mainly concerns differences in the type of stakeholders, where biofortification is a largely agriculture-based program, involving farmers, seed companies, and agricultural extension workers, while LSFF programs mainly involve producers of premixes and food manufacturers. Also, there are currently no national standards for biofortification programs, and biofortified foods have not yet penetrated markets at large scale. For these reasons, we decided to separate M&E frameworks and the selection of IMMT for biofortification and LSFF programs. Nevertheless, there is overlap between the frameworks when it comes to evaluating their effectiveness. Hence, it may be possible to integrate these two frameworks at the program outcome level (evaluation), while allowing them to differ at the input, activity and output level (monitoring).

We recommend further testing and revising the presented generic M&E framework with the nine indicators across LSFF programs within and between LMIC and implementing institutions, for subsequent harmonization of a global M&E framework for LSFF programs. A harmonized M&E framework will contribute to keeping track of fortification progress globally, exert positive pressure for effective LSFF implementation and advance the sharing of knowledge.

## 5. Discussion and conclusion

Based on this multi-method iterative study findings, we present here nine core indicators with their metrics, methods, and tools to be used for M&E of the effectiveness of LSFF programs in LMIC. The main strengths of the work presented here include (1) the use of a generic ToC framework for LSFF programming grounded in the available literature base, triangulated with tried-and-tested impact pathways of various LSFF programs implemented globally, and enriched with the perspectives of international experts; (2) identification of the most pertinent indicators used by LSFF programs; (3) selection of a core set of indicators with metrics, methods and tools, recommended for harmonization of M&E across LSFF programs and geographies; and (4) cross-checking of the compatibility of the generic ToC frameworks with their indicators in a local setting. The number of selected indicators has been kept to a minimum to facilitate efficient M&E practices in terms of cost, time and effort. We believe that this proposed set of core indicators provides the requisite information required for adaptive program management and results-oriented decision-making.

In the literature review, we limited ourselves to published reviews, assuming that they reflect the documented evidence to date. Also, our search strategy included literature published until 2020, thereby not considering potentially relevant literature published more recently. However, in an additional quick-scan of the literature, we did not find any reviews published up till August 2022 that would fulfill our inclusion criteria. Moreover, the grey literature was the main source of the core indicators finally selected. The discourse of the international experts aligned well with the key elements of a successful LSFF as described in our initial impact pathway (Supplementary Figure S1): (1) the selection of a food vehicle frequently consumed in sufficient quantities by large proportions of the population (in particular by vulnerable groups), so that fortification can make a meaningful contribution to micronutrient intake; (2) centralized production in a few large and well-establish industries; (3) sound internal QA/QC procedures mainstreamed into existing QA/QC protocols (i.e., not creating new QA/QC programs); (4) implementation of a simple and cost-effective enforcement system; and (5) effective, simplified, and sustainable assessment of coverage and consumption of fortified foods using HCES. However, the M&E framework proposed in this report has not yet been tested in context-specific programs, since we only explored perceptions towards the M&E framework, and particularly to the indicators, among program implementers in Nigeria and not in other settings. The relatively small number and diversity of key informants for LSFF programs from Nigeria (*n* = 11) was a limitation in this study, resulting in generalized perceptions that may not be shared by other program implementers in other contexts. Since participants represented different sectors and referred to different food vehicles, we were not able to extend our analysis to characterize specific LSFF programs, for example those of flour, oil, and salt. Also, some of the participants’ perceptions were based on a lack of familiarity with the indicators we presented to them and thus some responses lacked richness.

In conclusion, in this formative study, we formulated recommendations towards core indicators for comprehensive M&E of the performance and effectiveness of LSFF programs in LMIC. This work has resulted in a proposed set of nine core indicators and associated metrics, methods, and tools. This proposed set of core indicators can be used for further evaluation, harmonization, and integration in national and international protocols for M&E of LSFF program effectiveness.

## Author contributions

SR-M and FG wrote the first draft of the manuscript. SO, IB, EF, and KH developed the research proposal. SR-M fine-tuned the design of the study. TA, SR-M, and FG collected and analyzed the data. FS and AO took care of the interviews with key informants in Nigeria. SK and CL advised on specific aspects of the study. AM-B wrote the final version of the manuscript. All authors have read and approved the final version of the manuscript.

## Funding

Funding for the research was provided by Wageningen University & Research, as recipient of a Food Fortification Service (2FAS) grant from the European Union, Landell Mills, and the Global Alliance for Improved Nutrition (GAIN).

## Conflict of interest

KH was affiliated with Wageningen University & Research when she was involved in this project. Currently, she is employed by Pepsico Inc.

The remaining authors declare that the research was conducted in the absence of any commercial or financial relationships that could be construed as a potential conflict of interest.

## Publisher’s note

All claims expressed in this article are solely those of the authors and do not necessarily represent those of their affiliated organizations, or those of the publisher, the editors and the reviewers. Any product that may be evaluated in this article, or claim that may be made by its manufacturer, is not guaranteed or endorsed by the publisher.

## Footnotes

^1^ https://fortificationdata.org/

^2^ https://www.ffinetwork.org/

^3^ https://www.fantaproject.org/sites/default/files/Final-Arusha-Statement-on-Food-Fortification-Sep2015.pdf

^4^The Adult Male Equivalent (AME) has been developed by FAO to provide an expression of household food intake that accounts for the composition of the household and allows the direct comparison of food or energy intakes of households of different sizes and compositions. See Weissel and Dop (51).

^5^The MFI is an industry-led instrument that ranks food processing companies’ performance, introduced by Technoserve (https://mfi-ng.org/). It requires food industries to publicly report and track their performance data on food fortification, thereby providing a transparent way to measure and incentivize industry-wide progress on fortification.
