# Supplementary material for: A generic theory of change-based framework with core indicators for monitoring the effectiveness of large-scale food fortification programs in low- and middle-income countries
Source: Front Nutr. 2023 Jun 22;10:1163273. doi: 10.3389/fnut.2023.1163273 (PMC10324612; doi:10.3389/fnut.2023.1163273)

Supplementary material.

Search strategy for published literature

The building blocks and strings were developed based on the searching strategy of a systematic review performed by Keats *et al. [13]* and on a review protocol developed by Garcia-Casal *et al. [14].*

Building Blocks:

1 INDICATORS, METHODS, METRICS AND TOOLS

(monitor*[tw] OR evaluat*[tw] OR impact*[tw] OR assess*[tw] OR coverage[tw] OR indicator*[tw] OR method[tw] OR methods[tw] OR metric*[tw] OR tool*[tw] OR instrument*[tw] OR guideline*[tw] OR control*[tw] OR standard*[tw] OR measure*[tw] OR protocol*[tw] OR system*[tw])

2 NUTRITION POLICY

((“Health promotion"[Mesh] OR “Nutrition policy"[Mesh] OR “Health policy"[Mesh] OR “Mandatory programs"[Mesh] OR “Policy making"[Mesh] OR “Legislation, Food"[Mesh]) OR (nutrition*[tw] OR nutrition* program*[tw] OR food program*[tw] }?]

3 (BIO)FORTIFICATION AND FORTIFICATION

((“Food, fortified"[Mesh] OR "Biofortification"[Mesh]) OR (industrial* food fortif*[tw] OR industrial* fortif*[tw] OR fortificat*[tw] OR Food fortif*[tw] OR Fortif* food*[tw] OR “Food fortification program*”[tw] OR “Enrich* food*”[tw] OR “food enrichment”[tw] OR Enriched crop*[tw] OR crop* enrichment[tw] OR nutrition* enhanc* food*[tw] OR nutrition* enhanc* crop*[tw] OR Biofortif*[tw] OR Biofortif* food*[tw] OR Biofortif* crop*[tw] OR crop* Biofortif*[tw] OR food Biofortif*[tw] OR “plant* breeding”[tw] OR germ plasm*[tw] OR germplasm*[tw] OR biologic* fortifi*[tw]))

4 CROPS AND FOODS NAMES

((“Food"[Mesh] OR “Food Supply"[Mesh] OR “Crops, Agricultural"[Mesh] OR “Flour"[Mesh] OR “Salts"[Mesh] OR “Fish products"[Mesh] OR “Soy Foods"[Mesh] OR "Edible Grain"[Mesh] or “Triticum"[Mesh] OR “Zea mays"[Mesh] OR “Oryza"[Mesh] OR “Dietary Carbohydrates"[Mesh] OR “Milk"[Mesh] OR “Bread"[Mesh] OR “Oils"[Mesh] OR “Beverages"[Mesh] OR “Yogurt"[Mesh] OR “Margarine"[Mesh] OR “Cheese"[Mesh] OR “Condiments"[Mesh] OR “Spices"[Mesh] OR “Dietary Fats"[Mesh] OR “Dairy Products"[Mesh]) OR (Food[tw] OR foods[tw] OR staple[tw] OR crop*[tw] OR food crop*[tw] OR Plant[tw] OR plants[tw] OR “Food Suppl*”[tw] OR “Agricultural Crop*”[tw] OR vegetable*[tw] OR fruit[tw] OR fruits[tw] OR "Grain*”[tw] OR cereal*[tw] OR Flour*[tw] OR Bread[tw] OR nut[tw] OR nuts[tw] OR “Dietary Fat*”[tw] OR fat[tw] OR fats[tw] OR fatty[tw] OR Oil*[tw] OR “Dairy Product*”[tw] or dairy[tw] OR Milk[tw] OR Yogurt*[tw] OR Yoghurt*[tw] OR Margarine[tw] OR Cheese[tw] OR Salts[tw] OR Salt[tw] OR Condiment*[tw] OR Spices[tw] OR sauce*[tw] OR sugar*[tw] OR “curry powder*”[tw] OR “bouillon cube*”[tw] OR “stock cube*”[tw] OR “broth cube*”[tw] OR “soy* sauce”[tw] OR “fish sauce”[tw] OR wheat*[tw] OR Triticum[tw] OR maize*[tw] OR corn[tw] OR “Zea mays”[tw] OR rice*[tw] OR Oryza[tw] OR banana*[tw] OR “Musa paradisiaca”[tw] OR carrot*[tw] OR “Daucus carota” [tw] OR cowpea*[tw] OR “Vigna unguiculata”[tw] OR pea[tw] OR peas[tw] OR “Pisum sativum”[tw] OR peanut*[tw] OR “Arachis hypogaea”[tw] OR yam*[tw] OR “Dioscorea”[tw] OR rye[tw] OR “Secale cereale”[tw] OR taro[tw] OR “Colocasia esculenta”[tw] OR oat*[tw] OR “Avena sativa”[tw] OR cassava[tw] OR “Manihot esculenta”[tw] OR soybean*[tw] OR soyabean*[tw] OR “Glycine max”[tw] OR barley[tw] OR “Hordeum vulgare”[tw] OR sorghum[tw] OR “Sorghum bicolor”[tw] OR breadfruit*[tw]

OR“Artocarpus altilis”[tw] OR millet[tw] OR “Pennisetum glaucum”[tw] OR chickpea*[tw] OR “Cicer arietinum”[tw] OR lentil*[tw] OR “Lens culinaris”[tw] OR teff[tw] OR “Eragrostis tef”[tw] OR potato*[tw] OR “Solanum tuberosum”[tw] OR bean*[tw] OR “Phaseolus vulgaris”[tw] OR arrowroot[tw] OR “Maranta arundinacea”[tw] OR “orange sweet potato”[tw] OR “orange-fleshed sweet potato”[tw] OR “sweet potato orange”[tw] OR OFSP[tw] OR “Ipomoea batatas”[tw]))

5 MICRONUTRIENTS

((“Micronutrients"[Mesh] OR “vitamin a”[Mesh] OR "beta Carotene"[Mesh] OR “vitamin e”[Mesh] OR “vitamin d”[Mesh] OR "Folic Acid"[Mesh] OR “Zinc”[Mesh] OR “iron”[Mesh] OR "Ferric Compounds"[Mesh] OR "Iodine"[Mesh] OR “Selenium"[Mesh] OR “Trace Elements"[Mesh]) OR (micro-nutrient*[tw] OR micronutrient*[tw] OR multi-nutrient*[tw] OR multinutrient*[tw] OR “vitamin a”[tw] OR “beta carotene”[tw] OR carotene[tw] OR carotenoid*[tw] OR retinol[tw] OR retinoid[tw] OR “retinyl palmitate”[tw] OR “beta-cryptoxanthin”[tw] OR “vitamin e”[tw] OR “vitamin d”[tw] OR “Folic Acid”[tw] OR folic*[tw] OR folate*[tw] OR pteroylglutamic*[tw] OR zinc[tw] OR iron[tw] OR ferrous*[tw] OR ferric*[tw] OR "Ferric Compound*"[tw] OR iodin*[tw] OR seleni*[tw] OR “Trace Elements*”[tw]))

6 POPULATION

(("Developing Countries"[Mesh]) OR (developing countr*[tw] OR developing nation*[tw] OR developing population*[tw] OR developing world[tw] OR less* developed countr*[tw] OR less* developed nation*[tw] OR less* developed population*[tw] OR less* developed world[tw] OR less* developed countr*[tw] OR under developed countr*[tw] OR under developed nation*[tw] OR under developed population*[tw] OR under developed world[tw] OR underdeveloped countr*[tw] OR underdeveloped nation*[tw] OR underdeveloped population*[tw] OR underdeveloped world[tw] OR middle income countr*[tw] OR middle income nation*[tw] OR middle income population*[tw] OR low income countr*[tw] OR low income nation*[tw] OR low income population*[tw] OR lower income countr*[tw] OR lower income nation*[tw] OR lower income population*[tw] OR underserved countr*[tw] OR underserved nation*[tw] OR underserved population*[tw] OR underserved world[tw] OR deprived countr*[tw] OR deprived nation*[tw] OR deprived population*[tw] OR deprived world[tw] OR poor* countr*[tw] OR poor* nation*[tw] OR poor* population*[tw] OR poor* world[tw] OR developing econom*[tw] OR less* developed econom*[tw] OR under developed econom*[tw] OR underdeveloped econom*[tw] OR middle income econom*[tw] OR low income econom*[tw] OR low gdp[tw] OR low gnp[tw] OR low gross domestic[tw] OR low gross national[tw] OR lower income econom*[tw] OR lower gdp[tw] OR lower gnp[tw] OR lower gross domestic[tw] OR lower gross national[tw] OR lmic[tw] OR lmics[tw] OR third world[tw] OR lami countr*[tw] OR transitional countr*[tw] OR Africa[tw] OR Asia[tw] OR Caribbean[tw] OR West Indies[tw] OR South America[tw] OR Latin America[tw] OR Central America[tw] OR Afghanistan[tw] OR Albania[tw] OR Algeria[tw] OR American Samoa[tw] OR Angola[tw] OR Armenia[tw] OR Armenian[tw] OR Azerbaijan[tw] OR Bangladesh[tw] OR Byelarus[tw] OR Byelorussian[tw] OR Belarus[tw] OR Belorussian[tw] OR Belorussia[tw] OR Belize[tw] OR Benin[tw] OR Bhutan[tw] OR Bolivia[tw] OR Bosnia[tw] OR Herzegovina[tw] OR Hercegovina[tw] OR Botswana[tw] OR Brazil[tw] OR Brasil[tw] OR Bulgaria[tw] OR Burkina Faso[tw] OR Burkina Fasso[tw] OR Upper Volta[tw] OR Burundi[tw] OR Urundi[tw] OR Cape Verde[tw] OR Cabo Verde[tw] OR Cambodia[tw] OR Khmer Republic[tw] OR Kampuchea[tw] OR Cameroon[tw] OR Cameroons[tw] OR Cameron[tw] OR Central African Republic[tw] OR Chad[tw] OR China[tw] OR Colombia[tw] OR Comoros[tw] OR Comoro Islands[tw] OR Comores[tw] OR Mayotte[tw] OR Congo[tw] OR Republic democratic of Congo[tw] OR Zaire[tw] OR Costa Rica[tw] OR Cote d'Ivoire[tw] OR Ivory Coast[tw] OR Cuba[tw] OR Djibouti[tw] OR French Somaliland[tw] OR Dominica[tw] OR Dominican Republic[tw] OR Ecuador[tw] OR Egypt[tw] OR Arab Republic of Egypt[tw] OR United Arab Republic[tw] OR El Salvador[tw] OR Equatorial Guinea[tw] OR Eritrea[tw] OR Ethiopia[tw] OR Fiji[tw] OR Gabon[tw] OR Gabonese Republic[tw] OR Gambia[tw] OR The Gambia[tw] OR Georgia Republic[tw] OR Georgian Republic[tw] OR Georgia[tw] OR Ghana[tw] OR Grenada[tw] OR Guatemala[tw] OR Guinea[tw] OR Guinea-Bissau[tw] OR

Guiana[tw] OR Guyana[tw] OR Haiti[tw] OR Honduras[tw] OR India[tw] OR Indonesia[tw] OR Iran[tw] OR Republic Islamic Iran[tw] OR Iraq[tw] OR Jamaica[tw] OR Jordan[tw] OR Kazakhstan[tw] OR Kazakh[tw] OR Kenya[tw] OR Kiribati[tw] OR Korea[tw] OR Democratic People's Republic of Korea[tw] OR Kosovo[tw] OR Kyrgyzstan[tw] OR Kirghizia[tw] OR Kyrgyz Republic[tw] OR Kirghiz[tw] OR Kirgizstan[tw] OR "Lao PDR"[tw] OR Laos[tw] OR Lebanon[tw] OR Lesotho[tw] OR Basutoland[tw] OR Liberia[tw] OR Libya[tw] OR Macedonia[tw] OR North Macedonia[tw] OR Madagascar[tw] OR Malagasy Republic[tw] OR Malawi[tw] OR Nyasaland[tw] OR Malaysia[tw] OR Malaya[tw] OR Malay[tw] OR Sabah[tw] OR Sarawak[tw] OR Mali[tw] OR Marshall Islands[tw] OR Mauritania[tw] OR Mauritius[tw] OR Agalega Islands[tw] OR Mexico[tw] OR Micronesia[tw] OR Moldova[tw] OR Moldovia[tw] OR Moldovian[tw] OR Mongolia[tw] OR Montenegro[tw] OR Morocco[tw] OR Ifni[tw] OR Mozambique[tw] OR Myanmar[tw] OR Myanma[tw] OR Burma[tw] OR Namibia[tw] OR Nauru[tw] OR Nepal[tw] OR Nicaragua[tw] OR Niger[tw] OR Nigeria[tw] OR Pakistan[tw] OR Papua New Guinea[tw] OR Paraguay[tw] OR Peru[tw] OR Philippines[tw] OR Philipines[tw] OR Phillipines[tw] OR Phillippines[tw] OR Romania[tw] OR Rumania[tw] OR Roumania[tw] OR Russia[tw] OR Russian[tw] OR USSR[tw] OR Soviet Union[tw] OR Union of Soviet Socialist Republics[tw] OR Rwanda[tw] OR Ruanda[tw] OR Samoa[tw] OR Samoan Islands[tw] OR Navigator Island[tw] OR Navigator Islands[tw] OR Sao Tome[tw] OR São Tomé[tw] AND Principe[tw] OR Senegal[tw] OR Serbia[tw] OR Montenegro[tw] OR Sierra Leone[tw] OR Solomon Islands[tw] OR Somalia[tw] OR South Africa[tw] OR South Sudan[tw] OR Sri Lanka[tw] OR Ceylon[tw] OR Saint Lucia[tw] OR St Luci[tw] OR Saint Vincent[tw] OR St Vincent[tw] OR Grenadines[tw] OR Sudan[tw] OR Suriname[tw] OR Surinam[tw] OR Swaziland[tw] OR Syria[tw] OR Syrian[tw] OR Tajikistan[tw] OR Tadzhikistan[tw] OR Tadjikistan[tw] OR Tadzhik[tw] OR Tanzania[tw] OR Thailand[tw] OR Timor Leste[tw] OR East Timor[tw] OR East Timur[tw] OR Togo[tw] OR Togolese Republic[tw] OR Tonga[tw] OR Tunisia[tw] OR Turkey[tw] OR Turkmenistan[tw] OR Turkmen[tw] OR Tuvalu[tw] OR Uganda[tw] OR Ukraine[tw] OR Uzbekistan[tw] OR Uzbek[tw] OR Vanuatu[tw] OR New Hebrides[tw] OR Venezuela[tw] OR Bolivariana Republic of Venezuela[tw] OR Vietnam[tw] OR Viet Nam[tw] OR West Bank[tw] OR Gaza[tw] OR Yemen[tw] OR Republic of Yemen[tw] OR Zambia[tw] OR Zimbabwe[tw] OR Rhodesia[tw]))

Combination of Building Blocks:

1) 1 AND 2 AND 3 AND 4 AND 5 AND 6

Supplementary figure 1. Generic logic model for large-scale food fortification programs. Adapted from the generic logic model for micronutrient interventions in public health nutrition from the WHO/CDC (11).

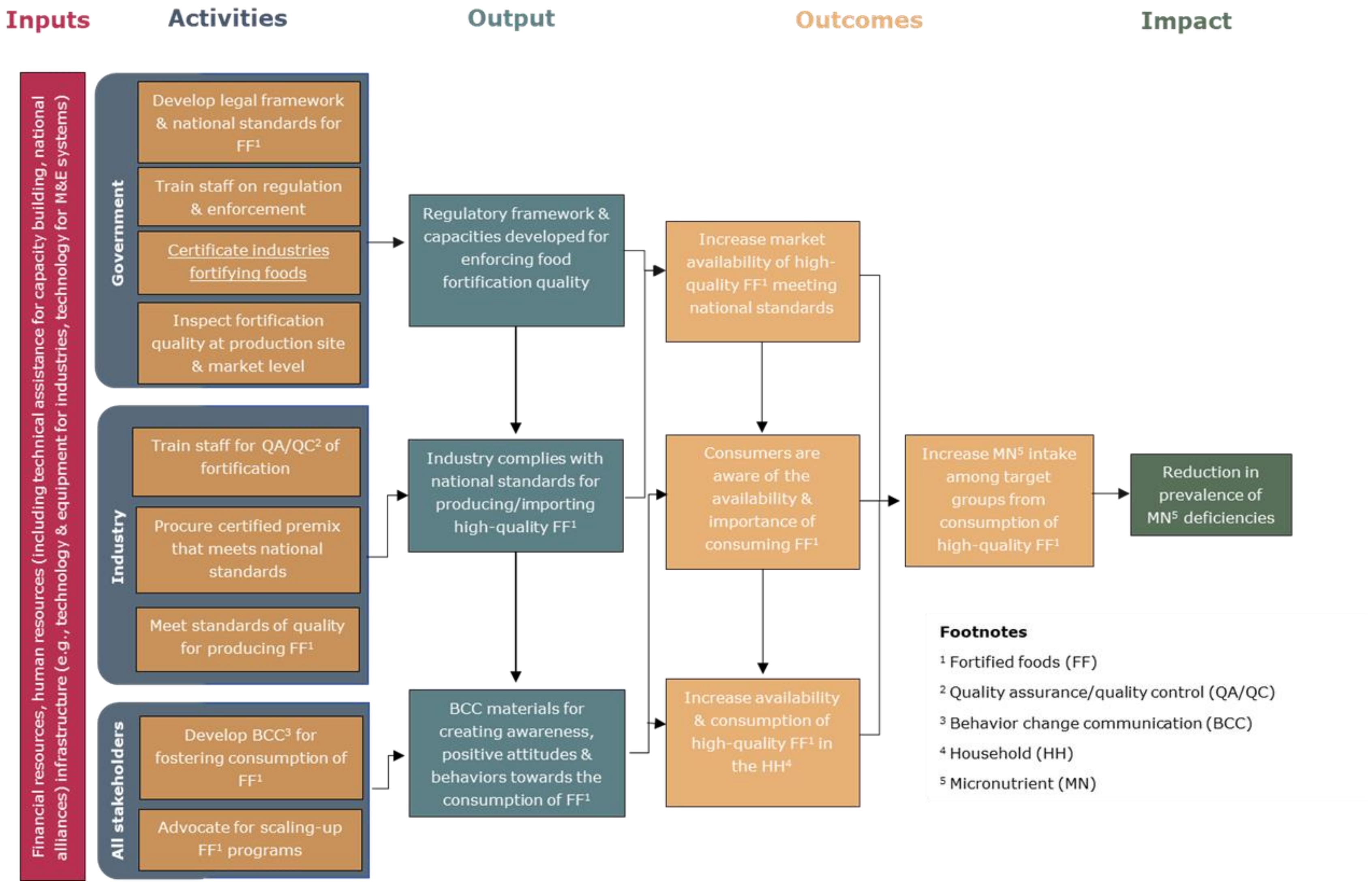

Supplement: Supplementary file 1 [file Data_Sheet_1.PDF]
